# Risk for partners’ depression and anxiety during pregnancy and up to one year postpartum: A longitudinal cohort study

**DOI:** 10.18332/ejm/148162

**Published:** 2022-06-22

**Authors:** Hafrún R. Finnbogadóttir, Eva K. Persson

**Affiliations:** 1 Department of Health and Caring Sciences, Faculty of Health and Life Sciences, Linnaeus University, Kalmar, Sweden; 2 Department of Health Sciences, Faculty of Medicine, Lund University, Lund, Sweden

**Keywords:** depression, pregnancy, anxiety, lifestyle, fathers, partners

## Abstract

**INTRODUCTION:**

Families may benefit from increased focus on partner emotional well-being during pregnancy and the perinatal period. Our aim was to explore the risk for depression and anxiety during pregnancy and one year postpartum in relation to partners’ self-reported health, sense of coherence, social support, and lifestyle factors.

**METHODS:**

This is a longitudinal cohort study using three questionnaires that were answered twice during pregnancy and at one year postpartum. Participants (n=532) were recruited between April 2012 and September 2013, and follow-up was between April 2012 and March 2015, in Sweden.

**RESULTS:**

In late pregnancy, 8.9% of the prospective partners were at high risk for depression and 8.3% one year postpartum. An increased risk for depression was found amongst those reporting ‘fair or very poor’ sexual satisfaction and those reporting ‘fair or very poor’ health during pregnancy and postpartum. High anxiety was reported by 10.8% during late pregnancy and 12.4% one year postpartum. Partners who were unemployed, had financial difficulties, and who scored low on a Sense of Coherence scale, showed significantly higher anxiety in late pregnancy and postpartum. Social support has a significant and positive impact concerning signs of depression and anxiety, both during pregnancy and postpartum.

**CONCLUSIONS:**

More than 10% of partners in this study showed depressive symptoms and anxiety, indicating a problem in need of attention by stakeholders. Strengthening social support is of greatest importance. It is time for the introduction of family-focused care aimed at prevention of depression and anxiety, and maintenance of family well-being.

## INTRODUCTION

The whole family may benefit from an increased focus on partner’s emotional well-being during pregnancy and the postpartum period^1,2^. Pregnancy and transition to parenting are critical life events that increase exposure to psychological stress and the possible onset of psychiatric disorders most often including depression in both parents^3,4^. However, maternal depression is more prevalent than paternal^4^. The most recent meta-analysis of Paternal Perinatal Depression (PPND) (defined as occurring during pregnancy and up to one year postpartum) included 74 studies from 22 countries covering five continents and showed paternal depression was present in about 8%^5^. PPND impacts negatively on family functioning, on couples’ relationships and on other family members’ health^6^. Studies^1,3,7^ have shown, that depression in fathers during the postnatal period was linked to adverse emotional and behavioral outcomes in their children. In men, depression manifests as fatigue, tiredness, self-criticism, touchiness, restlessness, and attacks of anger^8,9^. Depressive symptoms are often associated with anxiety and obsessive-compulsive disorder as well as a range of somatic symptoms along with alcohol and drug abuse, which can mask the main symptoms of PPND^8,9^. There is a high co-morbidity with maternal postpartum depression (PPD)^8,9^. Depressed men are more likely to show hyperactive or avoidant behavior, interpersonal conflicts and lower impulse control than depressed women^10^.

PPND is not an official psychiatric disorder and has no basis in any diagnostic manual unlike PPD which is codified in DSM-5^11^. PPND has not received the same attention as maternal PPD within the research community and the body of published studies is still growing. It is known, however, that a significant number of fathers experience anxiety and depression during the perinatal period^6^. Persson and Kvist^12^ showed a correlation between high levels of anxiety and increased risk for depression for both mother and father. An earlier publication, from the same project as the present study, showed that about one in ten prospective fathers/partners reported a high risk for depression and almost 9% had high anxiety in early pregnancy^13^. Sleeping difficulties and life-style factors such as smoking and the hazardous use of alcohol were reported in that study as determinants for risk for depression, as were socioeconomic factors such as being in financial distress and being unemployed^13^. It has been shown that a low Sense of Coherence (SOC), which reflects a coping capacity of individuals to deal with everyday life stressors during pregnancy, also increases the risk for depression during the perinatal period^13-15^. Social support has been shown to be of importance for mothers’ levels of postnatal depressive symptoms^14,16^. Therefore, we find it important to explore not only the risk for depression and anxiety but also to examine partners’ social support during the perinatal period. Previous studies on this important topic are relatively scarce.

The aim of this study was to explore the risk for depression and anxiety during pregnancy and up to one year postpartum in relation to partners’ self-reported health, Sense of Coherence, social support and lifestyle factors.

## METHODS

### Setting and participants

The present study has a longitudinal design with data from late pregnancy to one year postpartum and is a continuation from earlier published research from the same cohort^13^. The inclusion criteria were all partners (irrespective of gender) of pregnant women who were literate in Swedish or English.

The participants lived in an ethnically diverse region in southern Sweden and were recruited at 19 Antenatal Clinics (ANC) between April 2012 and September 2013 when accompanying their pregnant partner at registration in early pregnancy. Five of the ANCs are private care services and one ANC provided specialized care for complicated pregnancies and one unique facility which supports women with a history of drug abuse. Seven ANCs were situated in a multicultural, industrial city, four in a university city and six in smaller municipalities. Fathers/partners who satisfied the inclusion criteria were asked to participate and were recruited at the first visit to ANC at the same time as the pregnant women were recruited to a parallel study (completely independent), which has been reported earlier^17^. Further details regarding recruitment and setting are described in detail in an earlier published article^13^.

### Data collection

The data were prospectively collected between April 2012 and March 2015. A poster with information about the study was posted on the wall in the waiting room at the ANCs. Eighty-two midwives helped with the recruitment. If the partner was interested in taking part in the study, the midwife presented verbal and written information and written informed consent was obtained; the first (Q1) of three questionnaires was then completed^13^. The second (Q2) and the third (Q3) questionnaires were sent by regular mail to the postal address stated by the study participant on the consent form. Q2 was sent between gestational weeks 33–36 and Q3 approximately one year postpartum.

### The questionnaires

All data in this study are based on self-administered questionnaires: Q1, Q2 and Q3. The questionnaires consist of sociodemographic and lifestyle variables (employment and economy were covered in Q1 and Q3) and self-reported health (covered in all questionnaires) was measured using a question from the Short Form Survey (SF-36) instrument^18^, which measures an individual’s general state of health. Several other validated instruments were incorporated and are seen below. The number of questions in Q1 was 95, including thirteen questions from the instrument Sense of Coherence (SOC-13)^19^ which were used only in Q1. Q2 included 54 questions (SOC-13 and demographic questions excluded), and Q3 had 57 questions.

### Sociodemographic, lifestyle variables and self-reported health

The sociodemographic variables used in the present study were age, partner’s parity, education level, employment status, language spoken at home, financial difficulties and the SOC-13 instrument^19^. The lifestyle variables used are smoking, use of alcohol measured by Alcohol Use Disorders Identification Test (AUDIT)^20^, physical activity, sleeping difficulties and sexual satisfaction. Self-reported health is measured by the first question from the Short Form Survey (SF-36) instrument^18^.

### Instruments measuring lifestyle variables and selfreported health

Short Form Survey (SF-36) which is a validated and tested instrument, was used to measure self-reported health^18^. The first question in SF-36 was used and is as follows: ‘In general, would you say your health is (check one): 1) excellent, 2) very good, 3) good, 4) fair, or 5) poor?’.

AUDIT was used to measure drinking behavior^20^. AUDIT is a validated instrument for estimating risk use of alcohol. AUDIT consists of 10 items with statements on a 5-point Likert scale (0–4). The possible total score is between 0 and 40. For men, the cut-off points for ‘dangerous use’ of alcohol is ≥8 and for ‘hazardous use of alcohol’ ≥20^20^.

The SOC instrument^19^ was used to measure the salutogenic concept of sense of coherence. The SOC instrument tries to explain why some people become sick under stress while others stay healthy. The SOC instrument exists in a 29-item version and in a short version with 13 items. Regardless of age, gender, ethnicity and nationality, a high score is strongly related to perceived good health. The SOC-13 instrument, used in our study, is a reliable and valid instrument^19^.

### Outcome variables

Risk for depression in early and late pregnancy and at one year postpartum was measured using the Edinburgh Postnatal Depression Scale (EPDS)^21^. Anxiety was measured using the State version of State-Trait Anxiety Inventory (STAI) instrument^22^.

The EPDS instrument^21^ is a reliable and well validated instrument developed for measuring risk for postnatal depression and was originally developed for mothers. In recent years, the EPDS instrument has also been used for men^23,24^. The EPDS instrument consists of 10 items with statements on a 4-point Likert scale (0–3). Possible scores are between 0 and 30 and a higher score means a higher risk for postnatal depression^21^.

The STAI state instrument^22^ is a reliable and well validated instrument for measurement of anxiety ‘just now’. The STAI instrument exists in two versions, State and Trait. The Trait version measures anxiety in general and the State version measures anxiety ‘just now’. The STAI state version, used in our study, consist of 20 items and has statement on a 4-point Likert scale (1–4). Possible scores range between 20 and 80. Higher score means higher level of anxiety^22^.

### Classification of variables

Education level was dichotomized into two groups: ‘high’ (education including college /university) and ‘low’ (basic education and high school). Employment status was dichotomized as being ‘employed’ (including parental leave and studying) or ‘unemployed’ (including sick leave and on welfare benefit). The SOC score was dichotomized using the first quartile of the distribution as a cut-off value (SOC ≤64 and SOC >64)^25^. The SOC score was only computed for those who responded to all thirteen items.

The question for self-reported health was: ‘How is your health in general?’. The answers were dichotomized into ‘generally good’ and ‘fair or very poor’. Hazardous use of alcohol was calculated according to Saunders et al.^20^ AUDIT and dichotomized to ‘yes’ or ‘no’. In this study scores of ≥20 were used as the cut-off point. Sleeping difficulties, was dichotomized as ‘adequate sleep’ when answers were ‘no disturbance’ and ‘rarely any disturbance of sleep’ and ‘lack of sleep’ when answers were ‘disturbance sometimes or very often’. The category, sexual satisfaction, was dichotomized into two groups: ‘fair or very poor’ (if sex had not occurred in the past year or experienced sex as being ‘pretty or very poor’) and ‘fair or very good’ if reported as ‘good or very good’. Social support was accessed with four questions: 1) ‘Do you experience support from your partner during the pregnancy?’, with options ‘to a very great extent’, ‘to a great extent’, ‘to a small extent’, and ‘not at all’; 2) ‘At this stage of pregnancy, have you experienced the support from your mother?’; 3) ‘At this stage of pregnancy, have you experienced the support from your father?’. Question 2 and 3 had additionally an answer alternative to the first question for social support which was ‘not a life/not interest’; and 4) ‘Is there anyone else in your environment that you feel you are supported by right now?’, with responses ‘friends’, ‘siblings’, ‘colleague(s)’, ‘other’, and ‘nobody’.

In a previous study^13^, cut-off point of ≥10 on the EPDS scale for partners was chosen to represent the presence of symptoms of depression. Thus, in the present study, partners with an EPDS score <10 were considered at low risk for depression and partners with an EPDS score ≥10 were considered at high risk for depression. The total EPDS score was computed only for those who responded to all ten questions. In the current study, as in the first study from this material^13^, cut-off point for the STAI instrument was a score of ≥44 which indicated high anxiety, and normal anxiety was indicated by a score <44.

### Statistical analysis

Descriptive statistics were used for prevalence reporting. Cochrane’s Q-test was used to test for differences in partners’ risks for depression and anxiety at all three time points corresponding to the administration of Q1, Q2 and Q3. Chi-squared analysis was used to explore possible differences between partners with low and high risk for depression and partners with normal and high anxiety levels for sociodemographic variables, self-reported health, lifestyle factors, SOC-13, and social support. Friedman’s test was used to determine if there was a significant difference in perceived ‘social support’ from baseline in early pregnancy to one year postpartum (Q1–Q3). Correlation tests with Spearman’s correlation (ρ) were performed on the risk for depression (the EPDS instrument) and anxiety (the STAI state instrument). A weak relationship was defined as 0.1–0.3, a moderate as 0.3–0.5 and a strong relationship as >0.5. Statistical significance was accepted at p<0.05. The statistical analyses were performed using the Statistical Package for Social Sciences (SPSS) version 27.0 for Windows.

### Ethical considerations

According to the principles laid out by the World Medical Association Declaration of Helsinki, the research carried out in this study was justified^26^. The participants were given full information about the content of the study and informed written consent was obtained before participation. Approval was given by the Regional Ethical Review Board of Southern Sweden (Date and number: 2012/34).

## RESULTS

**Figure 1:**
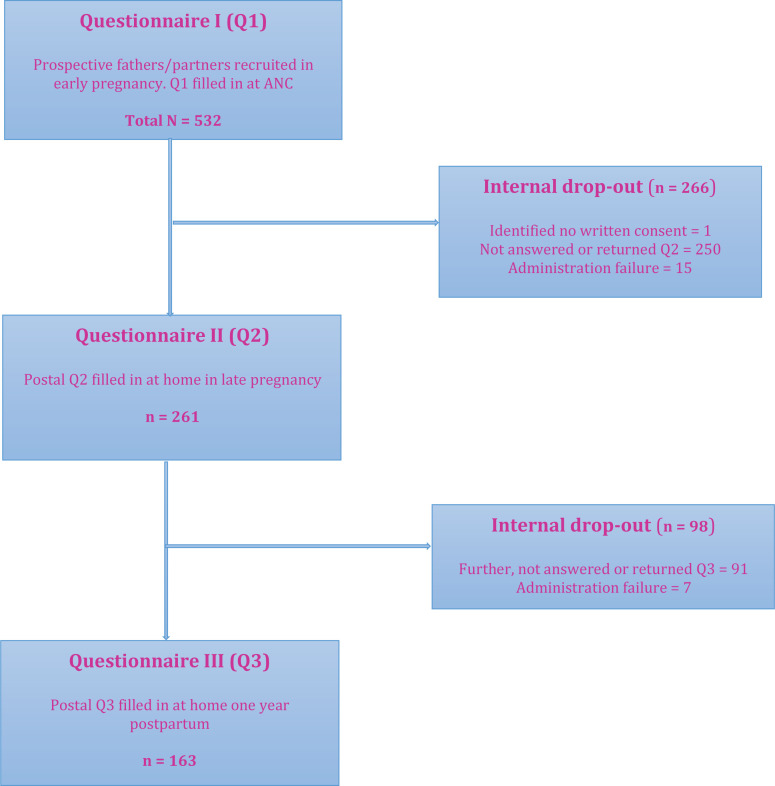
Flowchart over recruitment and received answers from questionnaires (Q1–Q3) and internal drop-out

Of a total cohort of 532 partners (only three were females) who participated in the previously reported study at baseline^13^; the response rate was 49% for Q2 in late pregnancy and 31% for Q3 at one year postpartum (Figure 1). The final drop-out was almost 69% from baseline. Drop-out mostly consisted of returned questionnaires because the recipients were no longer at their given address or the questionnaire was not returned at all.

Drop-out analysis showed that those who completed the study by answering Q3 had a statistically higher level of education, were employed, had no financial distress, spoke Swedish at home, and were non-smokers and alcohol users (Table 1).

**Table 1:** Sociodemographic differences between drop-out and partners who remained throughout the study and answered Q3 (N=532)*

*Characteristics*	*Total n (%)*	*Drop-out n (%)*	*Answered Q3 n (%)*	*p^a^*
	532 (100)	372 (69.9)	161 (30.1)	
**Age** (years)				0.136
18–25	61 (11.5)	48 (12.9)	13 (8.1)	
26–34	180 (33.8)	125 (33.6)	55 (34.4)	
31–35	188 (35.3)	135 (36.3)	53 (33.1)	
≥ 36	103 (19.4)	64 (17.2)	39 (24.4)	
**Parity**				0.478
Primiparae	194 (80.5)	102 (82.3)	194 (80.5)	
Multiparae	47 (19.5)	22 (17.7)	47 (19.5)	
**Language spoken at home**				**<0.001**
Swedish	456 (87.0)	308 (83.5)	148 (95.5)	
Foreign language	68 (13.0)	61 (16.5)	7 (4.5)	
**Education level**				**0.016**
High	335 (63.0)	222 (59.7)	113 (70.6)	
Low	197 (37.0)	150 (40.3)	47 (29.4)	
**Employment status**				0.073
Employed	484 (91.0)	333 (89.5)	151 (94.4)	
Unemployed	48 (9.0)	39 (10.5)	9 (5.6)	
**Financial distress**				**<0.001**
No	347 (65.2)	223 (59.9)	124 (77.5)	
Yes	185 (34.8)	149 (40.1)	36 (22.5)	
**Smoking/snuffing**				**0.005**
No	475 (90.6)	325 (88.3)	150 (96.2)	
Yes	49 (9.4)	43 (11.7)	6 (3.8)	
**Use of alcohol**				**0.003**
No	60 (11.5)	52 (14.1)	8 (5.1)	
Yes	464 (88.5)	316 (85.9)	148 (94.9)	
**Hazardous use of alcohol**				0.470
No	395 (82.3)	276 (83.1)	119 (80.4)	
Yes	85 (17.7)	56 (16.9)	29 (19.6)	

a Chi-squared p-value, statistical significance at p<0.05, two-tailed.

* Fathers/partners status in early pregnancy (Q1 or at baseline). Missing answers are between 7–298.

### Risk for depression

In late pregnancy, 8.9% of partners showed a high risk for depression, and at one year postpartum, 8.3% had a high risk for depression (Table 2). A Cochrane’s Q-test determined that there were no statistically significant differences in the proportion of partners with a high risk for depression at the three time-points (Q1=9.8%). The results from Q1 are presented in an earlier cross-sectional study^13^.

**Table 2:** Sociodemographic characteristics and sense of coherence of partners during late pregnancy and one year postpartum in relation to depression (N=532)

*Characteristics*	*Total at baseline n (%)*	*Q2 Low risk for depression n (%)*	*Q2 High risk for depression n (%)*	*P^a^ OR (95% CI)*	*Q3 Low risk for depression n (%)*	*Q3 High risk for depression n (%)*	*p^a^*
	532 (100)	234 (91.1)	23 (8.9)		143 (91.7)	13 (8.3)	
**Age** (years)*				0.372			0.483
17–25	61 (11.5)	19 (8.3)	3 (13.0)		11 (7.8)	1 (7.7)	
26–30	180 (33.8)	78 (33.9)	7 (30.4)		48 (34.0)	5 (38.5)	
31–35	188 (35.3)	83 (36.1)	11 (47.8)		45 (31.9)	6 (46.2)	
≥36	103 (19.4)	50 (21.7)	2 (8.7)		37 (26.2)	1 (7.7)	
Missing Q2–Q3=279/378							
Parity*				0.263			0.427
First-time parent	194 (80.5)	174 (82.5)	16 (72.7)		83 (80.6)	7 (70.0)	
Multi-parent	47 (19.5)	37 (17.5)	6 (27.3)		20 (19.4)	3 (30.0)	
Missing Q2–Q3=299/419							
Educational level*				0.060			0.444
High	335 (63.0)	172 (74.8)	13 (56.5)		101 (71.6)	8 (61.5)	
Low	197 (37.0)	58 (25.2)	10 (43.5)		40 (28.4)	5 (38.5)	
Missing Q2–Q3=279/378							
Employment status**				**0.022**			0.672
Employed	484 (91.0)	218 (94.8)	19 (82.6)	**3.8 (1.2–13.0)**	134 (95.5)	12 (92.3)	
Unemployed	48 (9.0)	12 (5.2)	4 (17.4)		7 (5.0)	1 (7.7)	
Missing Q2–Q3=279/376							
Language spoken at home*				0.823			0.404
Swedish	456 (87.0)	202 (89.4)	20 (90.9)		130 (94.9)	13 (100.0)	
Foreign language	68 (13.0)	24 (10.6)	2 (9.1)		7 (5.1)	0 (0.0)	
Missing Q2–Q3=284/389							
Financial distress**				**0.041**			
No	347 (65.2)	175 (76.1)	13 (56.5)	**2.4 (1.0–5.9)**	113 (80.1)	8 (61.5)	
Yes	185 (34.8)	55 (23.9)	10 (43.5)		28 (19.9)	8 (38.5)	
Missing Q2–Q3=279/378							
Sense of coherence score*				**0.023**			0.093
High	397 (79.1)	186 (84.2)	15 (65.2)	**2.8 (1.2–7.2)**	116 (85.3)	8 (66.7)	
Low	105 (20.9)	35 (15.8)	8 (34.8)		20 (14.7)	4 (33.3)	
Missing Q2–Q3=295/391							

a Chi-squared p-value, statistical significance at p<0.05, two-tailed. Bold indicates significant values.

* Only asked in Q1.

** Asked in Q1 and Q3.

### Associations between risk for depression and lifestyle variables and self-reported health

Partners with a high risk for depression belonged significantly more often to the group of unemployed and the group who reported financial difficulties, during late pregnancy but not one year postpartum. The group of unemployed at baseline was 9.0% and 4.3% one year postpartum (results not presented). During pregnancy, those who were unemployed measured low on SOC scores, and in late pregnancy when they answered Q2, they had a high risk for depression (Table 2).

Partners who reported their health as ‘fair or very poor’ at Q2 and Q3 had a statistically significantly higher risk for depression. There was a significantly higher risk for depression if the partners had sleeping difficulties both in late pregnancy and at one year postpartum (Table 3).

**Table 3:** Lifestyle factors among partners during late pregnancy and one year postpartum in relation to risk for depression (N=532)

*Characteristics*	*Total at baseline n (%)*	*Q2 Low risk for depression n (%)*	*Q2 High risk for depression n (%)*	*P^a^ OR (95% CI)*	*Q3 Low risk for depression n (%)*	*Q3 High risk for depression n (%)*	*p^a^*
	532 (100)	234 (91.1)	23 (8.9)		143 (91.7)	13 (8.3)	
**Self-reported health**				**<0.001**			**0.024**
Generally good	489 (96.1)	231 (98.7)	16 (69.6)	**3.1 (1.2–8.0)**	139 (92.7)	11 (84.6)	**6.3 (1.0–38.0)**
Fair or very poor	20 (3.9)	3 (1.3)	7 (30.4)		4 (2.8)	2 (15.4)	
Missing Q2–Q3=275/376		83 (36.1)	11 (47.8)		45 (31.9)	6 (46.2)	
**Smoking**				0.212			0.445
No	475 (90.6)	226 (96.6)	21 (91.3)		133 (95.7)	13 (100.0)	
Yes	49 (9.4)	8 (3.4)	2 (8.7)		6 (4.3)	0 (0.0)	
Missing Q2–Q3=275/380		174 (82.5)	16 (72.7)		83 (80.6)	7 (70.0)	
**Hazardous use of alcohol**				0.421			0.275
No	395 (82.3)	173 (79.0)	15 (71.4)		121 (93.1)	11 (84.6)	
Yes	85 (17.7)	46 (21.0)	6 (28.6)		9 (6.9)	2 (15.4)	
Missing Q2–Q3=292/389	335 (63.0)	172 (74.8)	13 (56.5)		101 (71.6)	8 (61.5)	
**Physical activity**				0.112			0.499
Yes	278 (53.0)	132 (56.4)	9 (39.1)		69 (48.3)	5 (38.5)	
No	247 (47.0)	102 (43.6)	14 (60.9)		74 (51.7)	8 (61.5)	
Missing Q2–Q3=275/376		218 (94.8)	19 (82.6)	3.8 (1.2–13.0)	134 (95.5)	12 (92.3)	
**Sleeping difficulties**				**0.013**			**<0.001**
Adequate sleep	393 (75.0)	218 (93.2)	18 (78.3)	**1.2 (1.0–1.5)**	132 (92.3)	8 (61.5)	**7.5 (2.0–27.0)**
Lack of sleep	131 (25.0)	16 (6.8)	5 (21.7)		11 (7.7)	5 (38.5)	
Missing Q2–Q3=275/376	456 (87.0)	202 (89.4)	20 (90.9)		130 (94.9)	13 (100.0)	
**Sexual satisfaction**				**0.027**			0.118
Fair or very good	465 (88.9)	181 (77.4)	13 (56.5)	**2.6 (1.1–6.3)**	116 (82.9)	12 (100.0)	
Fair or very poor	58 (11.1)	53 (22.6)	10 (43.5)		24 (17.1)	0 (0.0)	
Missing Q2–Q3=275/380							

a Chi-squared p-value, statistical significance at p<0.05, two-tailed. Bold indicates significant values.

### Anxiety

During late pregnancy, 10.8% of the partners reported high anxiety and 12.4% at one year postpartum. A Cochrane’s Q-test showed that there was no statistically significant difference between partners’ high level of anxiety at the three time points for the questionnaires (Q1=8.9%).

### Associations between risk for anxiety and lifestyle variables and self-reported health

Partners, with low education level were unemployed, had financial difficulties and scored low on SOC scale, reported statistically significantly more anxiety in late pregnancy as well at one year postpartum except for education level. Multi-parent with low scores on SOC scale one year postpartum also reported significantly more anxiety than those with a normal level of anxiety (Table 4).

**Table 4:** Sociodemographic characteristics and sense of coherence of partners during late pregnancy and one year postpartum in relation to anxiety (N=532)

*Characteristics*	*Total n (%)*	*Q2 Normal anxiety n (%)*	*Q2 High anxiety n (%)*	*P^a^ OR (95% CI)*	*Q3 Normal anxiety n (%)*	*Q3 High anxiety n (%)*	*P^a^ OR (95% CI)*
	532 (100)	222 (89.2)	27 (10.8)		141 (87.6)	20 (12.4)	
**Age** (years)*				0.997			0.302
17–25	61 (11.5)	18 (8.3)	2 (7.4)		12 (8.7)	0 (0.0)	
26–30	180 (33.8)	75 (34.4)	9 (33.3)		50 (36.2)	5 (25.0)	
31–35	188 (35.3)	79 (36.2)	10 (37.0)		43 (31.2)	9 (45.0)	
≥36	103 (19.4)	46 (21.1)	6 (22.2)		33 (23.9)	6 (30.0)	
Missing Q2–Q3=294/381							
Parity*				**0.029**			**0.006**
First-time parent	194 (80.5)	170 (83.7)	15 (65.2)	**2.7 (1.0–7.0)**	84 (82.4)	7 (50.0)	**4.7 (1.4–14.9)**
Multi-parent	47 (19.5)	33 (16.3)	8 (34.8)		18 (17.6)	7 (50.0)	
Missing Q2–Q3=313/423							
Education level*				**0.025**			0.308
High	335 (63.0)	165 (75.7)	15 (55.6)	**2.5 (1.1–5.6)**	95 (68.8)	16 (80.0)	
Low	197 (37.0)	53 (24.3)	12 (44.4)		43 (31.2)	4 (20.0)	
Missing Q2–Q3=294/381							
Employment status**				**<0.001**			**0.013**
Employed	484 (91.0)	208 (95.4)	21 (77.8)	**5.9 (2.0–18.0)**	137 (97.2)	17 (85.0)	**6.0 (1.2–29.0)**
Unemployed	48 (9.0)	10 (4.6)	6 (22.2)		4 (2.8)	3 (15.0)	
Missing Q2–Q3=294/381							
Language spoken at home*				0.293			0.922
Swedish	456 (87.0)	192 (89.7)	25 (96.2)		127 (95.5)	19 (95.0)	
Foreign language	68 (13.0)	22 (10.3)	1 (3.8)		6 (4.5)	1 (5.0)	
Missing Q2–Q3=299/386							
Financial distress**				**0.006**			**0.027**
No	347 (65.2)	167 (76.6)	14 (51.9)	**3.0 (1.3–6.9)**	115 (81.6)	12 (60.0)	**2.9 (1.1–7.9)**
Yes	185 (34.8)	51 (23.4)	13 (48.1)		26 (18.4)	8 (40.0)	
Missing Q2–Q3=294/381							
Sense of coherence score*				**<0.001**			**<0.001**
High	397 (79.1)	180 (86.1)	15 (57.7)	**4.55 (1.9–10.9)**	116 (87.9)	10 (52.6)	**6.5 (2.3–18.5)**
Low	105 (20.9)	29 (13.9)	11 (42.3)		16 (12.1)	9 (47.4)	
Missing Q2–Q3=304/388							

a Chi-squared p-value, statistical significance at p<0.05, two-tailed. Bold indicates significant values.

* Only asked in Q1.

** Asked in Q1 and Q3.

Partners who reported ‘fair or very poor’ health during late pregnancy also reported statistically significantly higher anxiety than those who reported their health as generally good. Smokers and those who were not physically active were significantly more anxious in late pregnancy than non-smokers and those who were physically active. Partners with sleeping difficulties both during pregnancy and postpartum had significantly higher anxiety levels. Also, partners who reported ‘fair or very poor’ sexual satisfaction during late pregnancy reported significantly higher anxiety levels (Table 5).

**Table 5:** Life-style factors among partners during late pregnancy and one year postpartum in relation to anxiety (N=532)

*Characteristics*	*Total n (%)*	*Pregnancy*			*Postpartum*		
		*Q2 Normal anxiety n (%)*	*Q2 High anxiety n (%)*	*P^a^ OR (95% CI)*	*Q3 Normal anxiety n (%)*	*Q3 High anxiety n (%)*	*P^a^ OR (95% CI)*
	532 (100)	222 (89.2)	27 (10.8)		141 (87.6)	20 (12.4)	
**Self-reported health**				**<0.001**			0.113
Generally good	489 (96.1)	220 (99.1)	20 (74.1)	**38.5 (7.5–19.7)**	137 (97.2)	18 (90.0)	
Fair or very poor	20 (3.9)	2 (0.9)	7 (25.9)		4 (2.8)	2 (10.0)	
Missing Q2–Q3=12/2							
**Smoking**				**0.005**			0.763
No	475 (90.6)	215 (96.8)	23 (85.2)	**5.3 (1.4–19.6)**	133 (96.4)	19 (95.0)	
Yes	49 (9.4)	7 (3.2)	4 (14.8)		5 (3.6)	1 (5.0)	
Missing Q2–Q3=12/5							
**Hazardous use of alcohol**							0.649
No	395 (82.3)	164 (79.6)	17 (68.0)		116 (92.1)	16 (88.9)	
Yes	85 (17.7)	42 (20.4)	8 (32.0)		10 (7.9)	2 (11.1)	
Missing Q2–Q3=30/19							
**Physical activity**				**0.001**			0.706
Yes	278 (53.0)	131 (59.3)	7 (25.9)	**4.6 (1.7–10.2)**	64 (46.7)	8 (42.1)	
No	247 (47.0)	90 (40.7)	20 (74.1)		73 (53.3)	11 (57.9)	
Missing Q2–Q3=13/7							
**Sleeping difficulties**				**<0.001**			**0.003**
Adequate sleep	393 (75.0)	209 (94.6)	19 (70.4)	**7.3 (2.6–0.1)**	129 (92.1)	14 (70.0)	**5.0 (1.6–15.7)**
Lack of sleep	131 (25.0)	12 (5.4)	8 (29.6)		11 (7.9)	6 (30.0)	
Missing Q2–Q3=13/3							
**Sexual satisfaction**				**0.045**			0.584
Fair or very good	465 (88.9)	170 (76.9)	16 (59.3)	**2.3 (1.0–5.2)**	115 (83.9)	15 (78.9)	
Fair or very poor	58 (11.1)	51 (23.1)	11 (40.7)		22 (16.1)	4 (21.1)	
Missing Q2–Q3=13/3							

a Chi-squared p-value, statistical significance at p<0.05, two-tailed. Bold indicates significant values. Questions asked in all three Q1–Q3.

### Correlation between father’s risk for depression and anxiety

Risk for depression (the EPDS) and anxiety (STAI state) showed a strong correlation over the course of the study.

### Experiences of social support during pregnancy and the postpartum

In early pregnancy, 90% of the total cohort reported that they experienced social support to a ‘high extent’ from their pregnant partner. In late pregnancy this was 91.9%, and 93.5% at one year postpartum. Experiences of social support to a ‘high extent’ from their own mother increased by approximately 10% from early pregnancy to one year postpartum (Q1=58.7%, Q2=69.3%, Q3=68.3%). The fathers/partners experienced their own father’s social support also to a ‘high extent’ and this increased by almost 12% from early pregnancy to one year postpartum (Q1=43.2%, Q2=56.4%, Q3=55.0%). Also, friends, siblings, colleagues and significant others were reported as providing social support both during pregnancy and postpartum (Q1=83.7%, Q2=87.5%, Q3=85.7%). Friedman’s test showed significant differences, between the three time points of administration of the questionnaires (Q1-Q3), of the extent fathers/partners reported experiencing social support from their own mother (p≤0.001) and father (p=0.020).

### Received social support during pregnancy and postpartum in relation to risk for depression and anxiety

Those fathers/partners who received social support to a higher extent from their pregnant partner in early and late pregnancy (Q1 and Q2) had a lower risk for depression (p=0.001, p=0.002, respectively) as well as a lower risk for anxiety (p=0.001, p=0.002, respectively). Also, those who received social support from their own mother in early pregnancy, as well as one year postpartum, had a lower risk for depression (p=0.018, p=0.002, respectively) and lower risk for anxiety one year postpartum (p=0.021). Those fathers/partners who received social support from their own father had lower anxiety in late pregnancy (p=0.045). If reviewed, social support to a higher extent from significant others the fathers/partners had significantly lower anxiety in early pregnancy and one year postpartum (p=0.004, p=0.017, respectively).

## DISCUSSION

An important finding in the present study was that during late pregnancy almost 8% and at one year postpartum almost 9% of partners, exhibited a high risk for depression. This result is in line with the most recent meta-analysis of PPND^5^. In our first report from the same cohort the figure was 10%^13^. The analyses showed no significant differences in partner’s risk for depression at the three different time points (Q1, Q2 and Q3). Our findings point to a need for directed support from the community for approximately one in ten expectant or new partners. This group of expectant partners had a less favorable socioeconomic situation (unemployed with financial difficulties) and scored low for SOC during pregnancy (although not one year postpartum), which is strongly related to poorer perceived health and which in turn may impact harmfully on other family members^6^. Fathers’ depression has been shown to be associated with emotional and behavioral consequences in their children^1,3,7^. In early pregnancy 9% were unemployed whereas at one year postpartum only 4.3% were unemployed. These lower figures represent dropouts from the study and may be one possible reason for significantly lower figures than we expected for anxiety and symptoms of depression one year after childbirth. This vulnerable group of partners who continued participation to Q3 reported poor health and had sleeping difficulties both during pregnancy and up to one year postpartum. The longitudinal design of the present study may in part be responsible for the high drop-out rates at Q2 and Q3 and therefore we conclude that there is a high hidden statistic since we were not successful in reaching those who were feeling worst and therefore there is a high likelihood of selection bias. How to reach this most vulnerable group is an unanswered question.

There is also a relationship between paternal and maternal depression^9,27^. Maternal mental health problems are considered as a major public health challenge worldwide^28^ with prevalence rates of perinatal depression reported to be between 10% and 20%^4,29^, impacting the health of the whole family^1^. A systematic review, meta-analysis and meta-regression of 291 studies from 56 countries showed a global prevalence of maternal postpartum depression (PPD) was 17.7% with significant heterogeneity across nations^30^. The primary risk factor for paternal depression has been shown to be maternal depression^5^; father’s history of severe depression or anxiety is also contributing factor. There is an urgent need for healthcare providers to reach out to those families who are in great need of support perinatally. Focus and resources should be directed to the most vulnerable families. Therefore, we propose that it is necessary, for the well-being of the family unit, to observe partners’ mental health in early pregnancy. During pregnancy, it may be difficult for the partners to ask their own questions, especially when the mother-to-be is present and therefore an exclusive meeting with the midwife both before and after birth should be offered to all partners. This is also supported by earlier research^13,31^. Screening of partner’s psychological health may prove to be as important as screening for maternal psychological health. Equality care for all cannot be the goal, but equity care is necessary. Priority and more resources must be offered to those who need it most. In the present study, we were not able to compare results from EPDS between partners because of ethical considerations. However, in clinical practice it is important to take in account both parents’ psychological health.

The results disclosed that up to 12% of fathers had high anxiety both during pregnancy and one year postpartum; both first-time fathers and multi-parents were anxious. This group also had a less favorable socioeconomic situation, poorer self-reported health as well as low scores on the SOC scale at both time-points. In the present study, anxiety and risk for depression showed a significantly strong correlation. As earlier discussed, paternal depression and anxiety can have both emotional and behavioral consequences for the offspring^7^. There is an urgency for action and revision of the way we work with families; results from the present and earlier research should be applied clinically. In dysfunctional families, it is often the children who suffer. The message is that it is important to identify both maternal and paternal risks for depression during pregnancy and the postpartum, because untreated perinatal depression and other mood disorders can have devastating consequences for the whole family^6^. Therefore, it is time for a paradigm shift with more family-focused care during pregnancy and during the postpartum period. Screening of fathers/partners for depression has increased in Sweden, but it is crucial that knowledge about the benefits of family-focused care is continuously relayed to caregivers.

Overall, our results showed that social support is a health factor and has significant importance concerning signs of depression and anxiety both during pregnancy and postpartum. Partners experienced increased social support from their own parents by almost 12% from early pregnancy to one year postpartum. As pregnancy is a matter for the family unit it is also important to address the partner’s social support in early pregnancy. Earlier research has shown that social support is of importance for the mothers well being^14,16^ but the research goes apart as regards social support for fathers^14,32^. There appears to be a need to further explore to what extent social support for fathers-to-be during the perinatal period is meaningful.

Sweden is considered to be the most gender-equal society in Europe, but many factors are weighed in the ranking, such as health, work, economy, violence, level of education, inequalities and political power^33^. In Sweden, the parents have equal right to parental leave until the child is 18 months old. Both parents are allowed 50% of the days, although three of the months are reserved for the parent who was not pregnant^34^. The remaining days can be transferred between the parents. Despite this, the taking of parental leave is still unequally distributed in Sweden. According to Central Organization of Salaried Employees, *‘Tjänstemännens centralorganisation’* [TCO]^34^, gender equality index, only 31.3% of the days were used by the partners last year. The main reason given for this is family economy, and the most socially vulnerable are absent in the group taking parental leave. It has been shown that fathers who did not take parental leave had a greater prevalence of PPND compared to fathers who did take leave^32^. This is an issue of high political priority^35^. The most vulnerable men with a low sense of coherence in our study were under-represented in answering the third questionnaire postpartum (Q3). In order to strengthen the sense of coherence among fathers/partners, it would be of importance to pay attention to this vulnerable group of men and guide them, already during pregnancy, to organized groups or arenas targeting fathers/partners-to-be.

### Strengths and limitations

The fact that the study was multi-centered and that data were collected prospectively may be considered as strengths of the study, as was the initial large cohort size. Also, the study included partners of both genders, although most were men. The use of validated instruments in the questionnaires is also a strength^18-22^. However, the drop-out rate that occurred between questionnaires Q1 and Q3 was considerable, with a final drop-out of more than two-thirds of the participants, which hampers the drawing of conclusions in our study. The findings may therefore be skewed. Those who constituted the drop-out group had less favorable sociodemographic and lifestyle factors. They had low levels of education, were unemployed, had financial difficulties, did not speak Swedish at home, and were smokers. Another limitation is that we do not know anything about the external drop-out group, but the internal drop-out group is mainly composed of fathers who had moved during pregnancy or the puerperium and were impossible to trace. This is a flaw in the study design; the collection of partners’ social security numbers might have mitigated this problem. A further limitation is the lack of information about fathers with pre-pregnancy depression. These fathers may require extra attention during the perinatal period, since their symptoms may cause extra vulnerability for the whole family. The fact that data collection was completed six years ago could be considered a weakness due to possible changes in society and healthcare during that time. However, in our point of view, these changes have not been so great as to influence our results.

The cut-off point for EPDS in the current study can also be a matter for discussion. Earlier studies have pointed to the fact that there may be a lower EPDS cut-off for fathers^23^. The reason is that the EPDS was developed for mothers and that some questions may measure mothers’ emotional reactions to a greater extent than the fathers’^23^ and therefore a lower cut-off for fathers may be recommendable. If a lower cut-off point had been used a higher rate of risk for depression would have been shown.

## CONCLUSIONS

At least one in ten fathers/partners has depressive symptoms and anxiety during pregnancy and up to one year postpartum, therefore it is vital to pay attention to the health and well-being of partners as well as the mother’s health. Received social support is of great importance to reduce symptoms of depression and anxiety. Societies need to act at a political level to strengthen vulnerable families. It is time for action to develop family-focused care to prevent and sustain the family’s well-being.

## Data Availability

The data supporting this research are available from the authors on reasonable request.
